# TM4SF1 Promotes Metastasis of Pancreatic Cancer via Regulating the Expression of DDR1

**DOI:** 10.1038/srep45895

**Published:** 2017-04-03

**Authors:** Jia-chun Yang, Yi Zhang, Si-jia He, Ming-ming Li, Xiao-lei Cai, Hui Wang, Lei-ming Xu, Jia Cao

**Affiliations:** 1 Department of Gastroenterology, Xinhua Hospital, School of Medicine, Shanghai Jiao Tong University, 1665 Kongjiang Road, Shanghai, 200092, China

## Abstract

Transmembrane-4-L-six-family-1(TM4SF1), a four-transmembrane L6 family member, is highly expressed in various pancreatic cancer cell lines and promotes cancer cells metastasis. However, the TM4SF1-associated signaling network in metastasis remains unknown. In the present study, we found that TM4SF1 affected the formation and function of invadopodia. Silencing of TM4SF1 reduced the expression of DDR1 significantly in PANC-1 and AsPC-1 cells. Through double fluorescence immuno-staining and Co-immunoprecipitation, we also found that TM4SF1 colocalized with DDR1 and had an interaction with DDR1. In addition, upregulating the expression of DDR1 rescued the inhibitory effects of cell migration and invasion, the expression of MMP2 and MMP9 and the formation and function of invadopodia when TM4SF1 silenced. In pancreatic cancer tissues, qRT-PCR and scatter plots analysis further determined that TM4SF1 had a correlation with DDR1. Collectively, our study provides a novel regulatory pathway involving TM4SF1, DDR1, MMP2 and MMP9, which promotes the formation and function of invadopodia to support cell migration and invasion in pancreatic cancer.

Pancreatic cancer is the fifth most common cause of cancer-related deaths in the United States, for which 5-year survival rate is 7%^1^. Although some progress has been made with regard to the clinical diagnose and the treatment methods, the mortality rate remains high. Tumor progression and metastasis are the major causes of mortality with pancreatic cancer patients^2^,^3^. Therefore, it is an urgent need to investigate the metastasis-related gene and to identify novel diagnostic methods and therapeutic targets in pancreatic cancer.

Transmembrane 4 L six family member 1 (TM4SF1) is a member of the L6 family, which is localized to the plasma membrane and is enriched in TM4SF1 enriched microdomains (TMED) that anchor nanopodia to regulate cell movement^4^. Recently, there have been an increasing number of studies on TM4SF1 in various malignant cancers, including liver cancer, breast cancer and colorectal cancer^5^,^6^,^7^. Our previous studies found that TM4SF1 was highly expressed in human pancreatic cancer tissues and various pancreatic cancer cell lines. And the expression of TM4SF1 was associated with pancreatic cancer metastasis and gemcitabine resistance *in vitro* and *in vivo*^8^,^9^. In PANC-1 and AsPC-1, TM4SF1 mediated the expression and activities of matrix metallopeptidase 2 (MMP2) and matrix metallopeptidase 9 (MMP9) which were the major regulators of extracellular matrix (ECM) degradation to increase the cells migration and invasion. Furthermore, TM4SF1 served as a molecular organizer that interacted with myosin-10 and β-actin involved in filopodia formation to mediate cell motility and directional migration. Also, TM4SF1 was necessary for the formation of the special projections that termed nanopodia^10^. These studies suggested that TM4SF1 played an essential role on the tumorigenesis and progression of pancreatic cancer.

Discoidin Domain Receptor 1 (DDR1), a subfamily of receptor tyrosine kinases (RTKs), is critical for cancer cell adhesion, proliferation and differentiation, cell migration and invasion. DDR1 is considered as a potential therapeutic target to design inhibitors to inhibit the proliferation of cells expressing high levels of DDR1 such as A549, MDA-MB-435, MCF-7 and HCT116 cell lines^11^,^12^,^13^. Moreover, DDR1 colocalizes with linear invadosomes which correlate with the ability to metastasize in cancer cells and may regulate their formation and the ability of matrix degradation through Tuba and Cdc42^14^. In pancreatic cancer, the expression of DDR1 is significantly higher and is related with poor prognosis in patients by the retrospective study^15^.

Both TM4SF1 and DDR1 are overexpressed in pancreatic cancer and related with metastasis development. And the recent study of multi-organ site metastatic reactivation in breast cancer demonstrated that TM4SF1 coupled DDR1 to activate PKCα to promote the signaling of JAK2/STAT3-mediated transcription of cancer stem cell genes^16^. Thus, we hypothesized that TM4SF1 might collaborate with DDR1 involved in the formation of invadopodia which had the ability to degrade ECM to regulate pancreatic cancer metastasis.

## Results

### TM4SF1 is necessary for invadopodia formation and function

**Figure 1:**
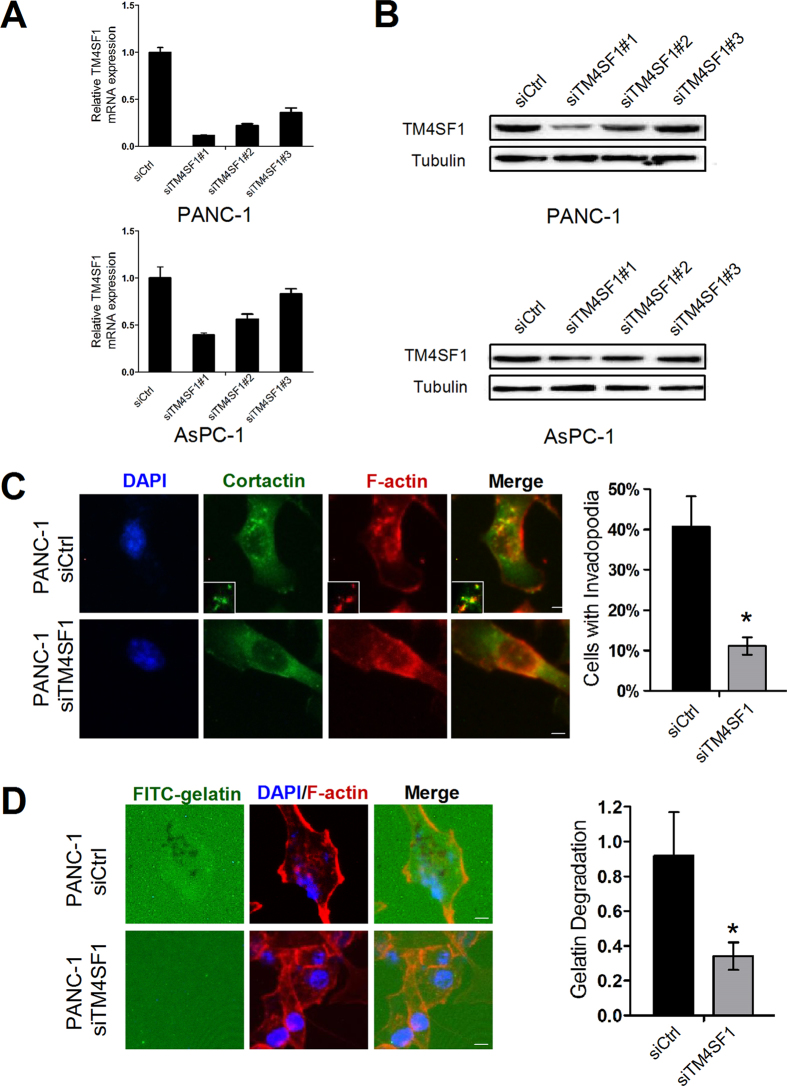
TM4SF1 is necessary for invadopodia formation and function. (**A**,**B**) qRT-PCR and Western blot were used to detect the expression of TM4SF1 when PANC-1 and AsPC-1 cells were transfected with siCtrl or siTM4SF1. (**C**) PANC-1 cells transfected with siCtrl or siTM4SF1 were stained with DAPI, phalloidin, and cortactin. Quantification of percentage of cells with invadopodia decreased after silencing TM4SF1 in PANC-1. *P < 0.05. (**D**) PANC-1 cells transfected with siCtrl or siTM4SF1 were plated on FITC-conjugated gelatin. F-actin was stained with phalloidin. Areas of gelatin degradation appeared as black areas. Quantification of FITC-gelatin degradation in PANC-1 cells treated with siTM4SF1 significantly decreased. *P < 0.05.

As previously described, the abilities of migration and invasion in PANC-1 and AsPC-1 cells decreased when TM4SF1 suppressed. Here, we wondered whether TM4SF1 expression might affect the formation and function of pancreatic cancer cell invadopodia. We first transiently transfected PANC-1 and AsPC-1 with siCtrl or three siTM4SF1. Transfection efficiency was evaluated by qRT-PCR and western blot. According to the results, we chose siTM4SF1#1 to decrease the expression of TM4SF1 (Fig. 1A,B and S1). To determine the presence of invadopodia, we stained cells with DAPI, phalloidin, and cortactin. The results suggested that over 40% of PANC-1 siCtrl cells contained invadopodia compared with approximately 11% of PANC-1 siTM4SF1 cells (Fig. 1C). Next, we examined the function of TM4SF1-induced invadopodia and found that silencing of TM4SF1 decreased matrix degradation index by approximately 63% compared with the control (Fig. 1D). These data demonstrated that the expression of TM4SF1 affected the formation and function of invadopodia.

### TM4SF1 regulates DDR1 expression and interacts with DDR1

**Figure 2:**
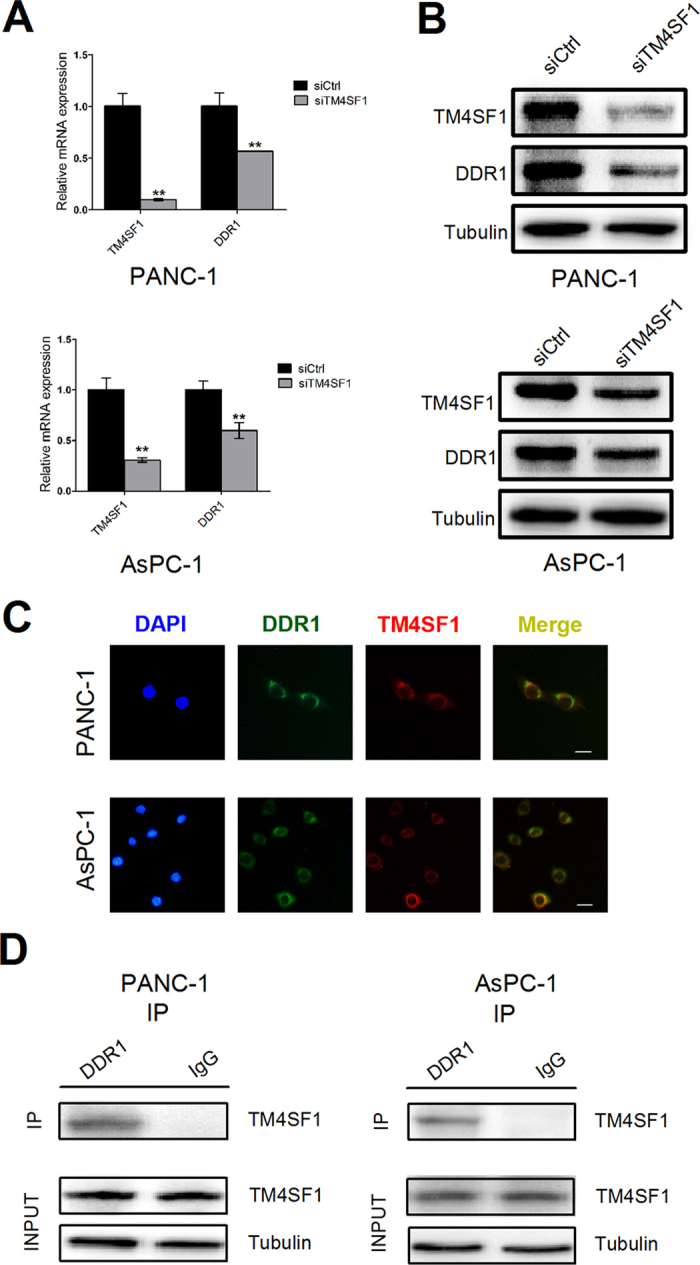
TM4SF1 regulates DDR1 expression and interacts with DDR1. (**A**,**B**) the mRNA and protein expression levels of TM4SF1 and DDR1 were detected by qRT-PCR and western bolt analysis. The mRNA and protein expression levels of DDR1 decreased significantly in PANC-1 and AsPC-1 after transfected with siTM4SF1. **P < 0.01. (**C**)TM4SF1 colocalized with DDR1 in PANC-1 and AsPC-1 using double fluorescence immunostaining. (**D**) co-IP assays showed an interaction between TM4SF1 and DDR1.

To examine whether TM4SF1 regulated DDR1 expression in pancreatic cancer cell lines, we transfected siRNA into cells and found that the down-regulation of TM4SF1 could decrease the mRNA and protein expression levels of DDR1 in PANC-1 and AsPC-1 (Fig. 2A,B). Using double fluorescence immuno-staining, we investigated that TM4SF1 colocalized with DDR1 in PANC-1 and AsPC-1 (Fig. 2C). In addition, co-IP assays showed an interaction between TM4SF1 and DDR1 (Fig. 2D).

### TM4SF1-induced migration and invasion requires DDR1

**Figure 3:**
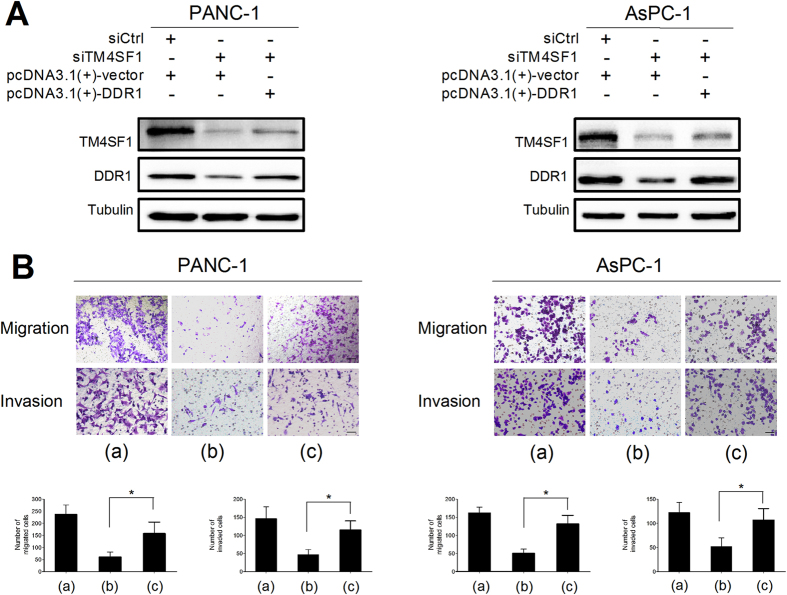
TM4SF1-induced migration and invasion requires DDR1. (**A**) western blot showed the protein expression of TM4SF1 and DDR1. The upregulation of DDR1 expression attenuated the loss of DDR1 expression after TM4SF1 silenced. (**B**) Transwell assays showed that upregulating the expression of DDR1 rescued the inhibitory effects of migration and invasion after decreasing the expression of TM4SF1.*P < 0.05. (a) siCtrl + pcDNA3.1(+)-vector (b) siTM4SF1+pcDNA3.1 (+) -vector (c) siTM4SF1 + pcDNA3.1(+)-DDR1.

To further validate that TM4SF1 mediated pancreatic cancer cell migration and invasion by regulating DDR1, we increased the expression of DDR1 in TM4SF1 silenced cells and then observed the protein expression levels of TM4SF1 and DDR1 and the abilities of cell migration and invasion by western blot and Transwell assay. The immunoblot analysis results showed that silencing the expression of TM4SF1 decreased DDR1 expression, whereas the up-regulation of DDR1 attenuated the loss of DDR1 expression in TM4SF1 silencing cells (Fig. 3A). Also, silencing TM4SF1 in PANC-1 and AsPC-1 decreased the abilities of cell migration and invasion, whereas upregulating the expression of DDR1 rescued the inhibitory effects of migration and invasion after decreasing the expression of TM4SF1 (Fig. 3B).

### DDR1 over-expression rescues the inhibitory effects by TM4SF1 silenced

**Figure 4:**
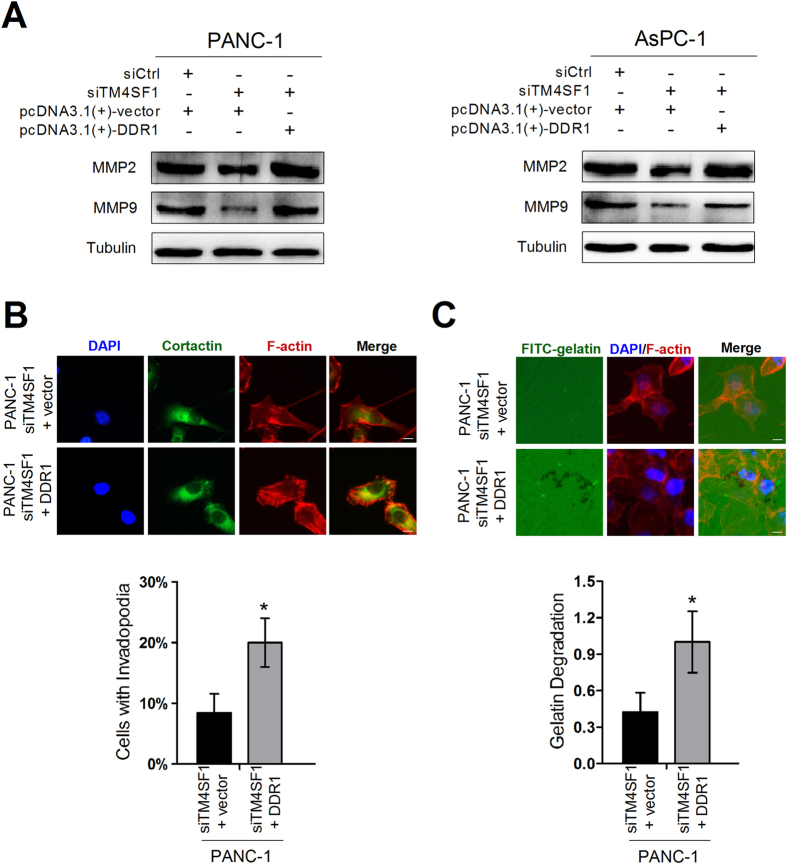
DDR1 over-expression rescues the inhibitory effects by TM4SF1 silenced. (**A**) western blot analysis showed that silencing TM4SF1 decreased the expression levels of DDR1, MMP2 and MMP9, whereas upregulating the expression of DDR1 rescued the decreased expression of MMP2 and MMP9. (**B**,**C**) upregulation of DDR1 expression levels rescued the inhibition of invadopodia forming and matrix degrading after TM4SF1 silenced. *P < 0.05.

Through siRNA and plasmids transfection, we found that silencing TM4SF1 decreased the expression of DDR1, MMP2 and MMP9, whereas up-regulation of DDR1 expression rescued the decreased expression of MMP2 and MMP9 (Figs 3A and 4A). pcDNA3.1-DDR1 plasmids were transfected in PANC-1/siTM4SF1 cells to overexpress DDR1 to determine whether TM4SF1 had an interaction with DDR1 to affect the formation and function of invadopodia. The results proved that overexpression of DDR1 significantly increased the ability of PANC-1/siTM4SF1 cells to form invadopodia (Fig. 4B) and degrade FITC-gelatin matrix (Fig. 4C). Tks5, an adaptor protein required for invadopodia formation, was also used to investigate the role of TM4SF1 and DDR1 in pancreatic cancer cells. The staining showed that suppression of TM4SF1 reduced the ability to form invadopodia in PANC-1. In contrast, overexpressing of DDR1 increased the number of cells with invadopodia (Figs S2A and S2B).

### TM4SF1 correlates with DDR1 expression in specimens of pancreatic cancer

**Figure 5:**
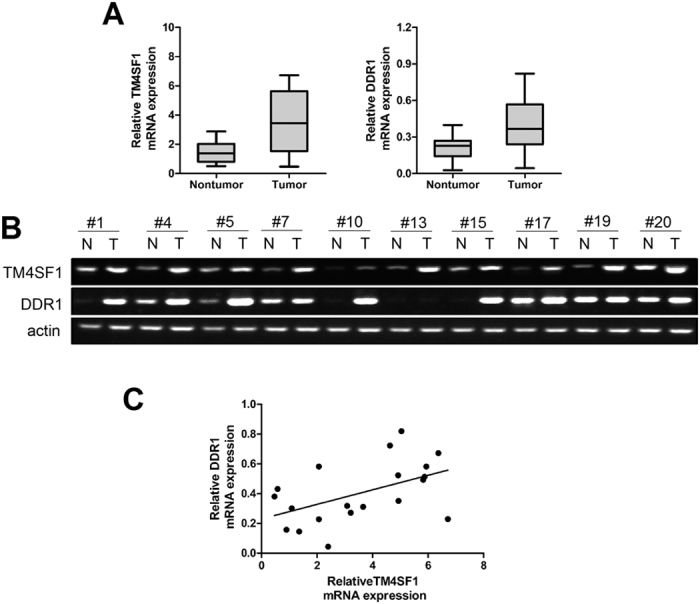
TM4SF1 correlates with DDR1 expression in specimens of pancreatic cancer. (**A**,**B**) TM4SF1 and DDR1 expression levels in twenty pairs of pancreatic cancer tissue samples were detected by qRT-PCR. TM4SF1 and DDR1 mRNA expression levels were both higher expressed in pancreatic cancer tissues compared with adjacent non-tumor samples. (**C**) scatter plots showed a positive correlation between TM4SF1 and DDR1 at mRNA expression level in pancreatic cancer.

We next investigated the role of TM4SF1 and its relationship with DDR1 in pancreatic cancer tissues, we measured the mRNA expression levels of TM4SF1 and DDR1 in 20 pairs of pancreatic cancer tissue samples. The mRNA expression levels were higher in pancreatic cancer tissues than in non-tumor paired-adjacent tissues (Fig. 5A,B). We further analyzed and plotted TM4SF1 mRNA expression levels against the levels of DDR1 in pancreatic cancer tissue. The results showed that there was an obvious positive correlation between TM4SF1 and DDR1 mRNA expression in tumor tissue samples (Fig. 5C).

## Discussion

Cancer metastasis is a complex multistep and highly regulated cascade which severely influences the effectiveness and prognosis of patients. Cancer cells disseminate from the original sites, degrade basement membrane (BM) and extracellular matrix (ECM), survive in the vascular system and eventually extravasate across the endothelium to colonize secondary sites and grow. Many studies have displayed that degradation of ECM is an initiated step in the process of invasion and metastasis^17^,^18^,^19^.

Pancreatic cancer is characterized by extensive desmoplastic reaction, resulting in tumor containing more non-tumor cells and extracellular matrix stroma^20^. As we known, invadopodia which are closely associated with ECM degradation that form the invasion mechanism of pancreatic cancer cells. MMP2, MMP9 and MT1-MMP are all enriched at the invadopodia that mediate ECM degradation to accelerate metastasis in cancer^21^,^22^. Our previous study found that *in vitro* experiments, silencing of TM4SF1 suppressed MMP2 and MMP9 expression and activation to reduce the migration and invasion in PANC-1 and AsPC-1^8^. Therefore, we focused on the molecular mechanism of TM4SF1 on invadopodia. In this study, we observed that silencing of TM4SF1 decreased the number of cells with invadopodia and matrix degradation index in PANC-1, indicating that TM4SF1 efficiently regulated the formation and function of invadopodia to degrade ECM during pancreatic cancer cell migration and invasion.

The report showed that extracellular portion of DDR1 was sufficient for collaboration with TM4SF1. Based on the structure, the discoidin-like domain or the ~50 amino acid-long proximal membrane segment of the ectodomain of DDR1 might interact with the extracellular loop connecting the third and fourth transmembrane of TM4SF1. And TM4SF1 coupled DDR1 in the process of metastasis in breast cancer cells to PKCα and enhanced JAK-STAT signaling^16^,^23^,^24^,^25^,^26^. According to these results, it would be interesting to see whether DDR1 involved in forming invadopodia of TM4SF1-expressing cells. Through experiment, we determined that silencing of TM4SF1 decreased the expression levels of DDR1 and TM4SF1 colocalized with DDR1 in PANC-1 and AsPC-1. The co-IP assays result and a positive correlation between TM4SF1 and DDR1 expression of pancreatic cancer tissues further provided the evidence of this interaction.

In addition, MMPs frequently overexpressed and correlated with invasive capacity and poor prognosis in various cancers such as breast, stomach, colon and prostate cancer. The ECM degrading ability of invadopodia was mostly due to MMPs, including MT1-MMP, MMP2 and MMP9. MMP inhibitor down-regulated ECM degradation and the formation of invadopodia, suggesting a positive feedback loop in which degradation products from MMP promoting new invadopodia formation^27^,^28^,^29^,^30^,^31^,^32^. Our study investigated that when overexpressed DDR1 in TM4SF1-silenced cells, the number of pancreatic cancer cells with invadopodia and the expression of MMP2 and MMP9 were increased. Also, upregulating the expression of DDR1 rescued the inhibitory effects of migration and invasion in TM4SF1-silenced cells. These results indicated that TM4SF1 on cell surface might collaborate with DDR1 to increase the formation of invadopodia and the expression of MMP2 and MMP9 which were crucial events in pancreatic cancer cell migration and invasion.

As we known, integrins are the key protein involved in the process of cell migration.TM4SF1 interacted with integrin α5 and integrin β1 subunits to mediate endothelial cell migration and promote cell-cell interaction^33^. DDR1 signaling and integrin signal transduction could not only be independent, but also had the opposite effect in a certain context. For example, in BxPC-3 cells, integrins activating FAK and DDR1 activating Pyk2 cooperated in collagen I-mediated epithelial to mesenchymal transition. On the contrary, DDR1 inhibited activators of transcription 1/3 and cell migration triggered by integrin α2β1 in collagen gels in MDCK cells^34^,^35^. In the previous study, using nilotinib to inhibit DDR1 kinase activity showed DDR1 kinase activity was not required for linear invadosome formation and activity^14^. In breast cancer cells, silencing of TM4SF1 did not inhibit the kinase activation of DDR1 on cell clustering, suggesting that DDR1 had its own ability^13^. Therefore, further studies in our laboratory will be focused on the function of TM4SF1, DDR1 and integrins in pancreatic cancer cells contacting with collagen.

Together, these results indicated that TM4SF1-induced invadopodia formation and function in pancreatic cancer cells were associated with the expression of DDR1. TM4SF1 may be used as a promising target for metastatic prediction and individualized drug therapy.

## Material and Methods

### Cell culture and Tissue Samples

The pancreatic cancer cell lines PANC-1 and AsPC-1 were purchased from Cell Bank of Type Culture Collection of Chinese Academy of Sciences (Shanghai, China). PANC-1 cells were cultured in DMEM medium supplemented with 10% fetal bovine serum (FBS), 100 U/ml penicillin and 100 μg/ml streptomycin. AsPC-1 cells were maintained in RPMI-1640 medium supplemented with 10% FBS as well as 100 U/ml penicillin and 100 μg/ml streptomycin. Cells were incubated in a humidified atmosphere of 5% CO_2_ at 37 ^o^C. Samples of human pancreatic cancer tissues and paired-adjacent tissues were obtained from the Department of Pathology and General Surgery at Xin Hua Hospital affiliated to Shanghai Jiao Tong University School of Medicine. All methods were approved by the research medical ethics committee of Xinhua Hospital Affiliated to Shanghai Jiaotong University School of Medicine and were carried out in accordance with the approved guidelines. Informed consent on the use of clinical specimens were obtained from all patients.

### RNA interference and plasmids transfection

Small interfering RNA (siRNA) against TM4SF1and control siRNA were purchased from Gene Pharma (Shanghai, China). The plasmids pcDNA3.1-DDR1 and empty vector plasmids were produced by Genechem (Shanghai, China). All transfections were performed using Lipofectamine 2000 according to the manufacturer’s instructions.

### RT-PCR and qRT-PCR

Total RNA was isolated from pancreatic cancer cells or tissues using Trizol reagent (Takara, Japan). And the RNA was reverse transcribed using cDNA Reverse Transcription Kit (Takara, Japan). RT-PCR and qRT-PCR were performed using a RT-PCR kit and a SYBR Green Premix Ex Taq II (Takara, Japan) according to the instructions.

### Western blot and Co-immunoprecipitation

Cells were collected and lysed in RIPA buffer (Beyotime, China) with 10 nM PMSF for 20 min, qualified and then performed for SDS-PAGE. The protein on the SDS gels was transferred to PVDF membranes. Antibodies against TM4SF1 (1:2000 dilution, Abcam, USA), DDR1 (1:1000 dilution, CST, USA), MMP2 (1:1000 dilution, Santa Cruz Biotechnology, USA) and MMP9 (1:500 dilution, Abcam, USA) were used to detect the protein level. β- tubulin (1:1000, Beyotime, China) was used as control to equal protein loading. The signals were detected by an enhanced chemiluminescence detection kit (Millpore, USA). For co-IP, PANC-1 and AsPC-1 cells at 80–90% confluence were washed with ice-cold DPBS three times before being lysed in IP lysis buffer. Then the lysates were incubated with antibodies overnight at 4 ^o^C. Protein A/G PLUS Agarose beads were added for 10 h at 4 ^o^C. The beads were collected and washed with lysis buffer for three times. The precipitated proteins were eluted and denatured in 2 × SDS loading buffer and analyzed by western blot.

### Immunofluorescence

PANC-1 and AsPC-1 cells were fixed in 4% paraformaldehyde at room temperature for 10 min, permeabilized with 0.1% Triton X-100 for 20 min and then blocked with 5% donkey serum. Cells were double-stained for TM4SF1 and DDR1 overnight at 4 ^o^C and then with secondary antibodies for 1 h at room temperature. In the invadopodia formation assay, cells were stained for cortactin or Tks5 overnight at 4 ^o^C and incubated with TRITC Phalloidin to detect F-actin for 30 min at room temperature. After washing three times in DPBS, the DAPI was used to costain the DNA^36^.

### *In situ* zymography

Coverslips were coated with FITC-gelatin (Invitrogen, USA), rinsed in DPBS and incubated at 37 ^o^C in culture medium for 2 h. Cells were seeded on coated coverslips and incubated for overnight before fixation and staining with actin. Images were taken at eight fields per sample. Gelatin degradation was quantified using ImageJ software. The final gel degradation index was the average percentage degradation obtained from the ten fields^36^,^37^.

### Cell migration and invasion assay

PANC-1 and AsPC-1 were plated in 6-well plates and transfected with siRNA or/and plasmid. Before conducting experiments, Transwell chambers were prepared. Cell migration and invasion were evaluated as described^36^,^38^. After 10 h of incubation, the migrated and invaded cells were fixed and stained with crystal violet and counted under a microscope at least eight randomly selected fields in each membrane.

### Statistical analysis

All data were expressed as the mean ± SD and analyzed using SPSS 19.0 software. Statistical analyses were performed by Student’s t-test and one-way ANOVA test. A P-value less than 0.05 was considered as statistically significant.

## Additional Information

**How to cite this article:** Yang, J.- *et al*. TM4SF1 Promotes Metastasis of Pancreatic Cancer via Regulating the Expression of DDR1. *Sci. Rep.* **7**, 45895; doi: 10.1038/srep45895 (2017).

**Publisher's note:** Springer Nature remains neutral with regard to jurisdictional claims in published maps and institutional affiliations.

## Supplementary Material

Supplementary Figures
