# Supplementary material for: TM4SF1 Promotes Metastasis of Pancreatic Cancer via Regulating the Expression of DDR1
Source: Sci Rep. 2017 Apr 3;7:45895. doi: 10.1038/srep45895 (PMC5377454; doi:10.1038/srep45895)

**TM4SF1 Promotes Metastasis of Pancreatic Cancer via Regulating the Expression of DDR1**

Jia-chun Yang, Yi Zhang, Si-jia He, Ming-ming Li, Xiao-lei Cai, Hui Wang, Lei-ming Xu*, and Jia Cao*

**Supplementary Figure S1. siTM4SF1#1 was chosen to decrease the expression of TM4SF1 for the further study.** The protein expression levels of TM4SF1 were detected when PANC-1 and AsPC-1 cells were transfected with siCtrl or siTM4SF1. Tubulin was used as a control for the expression levels. Data are presented as the mean ± SD (n=3).


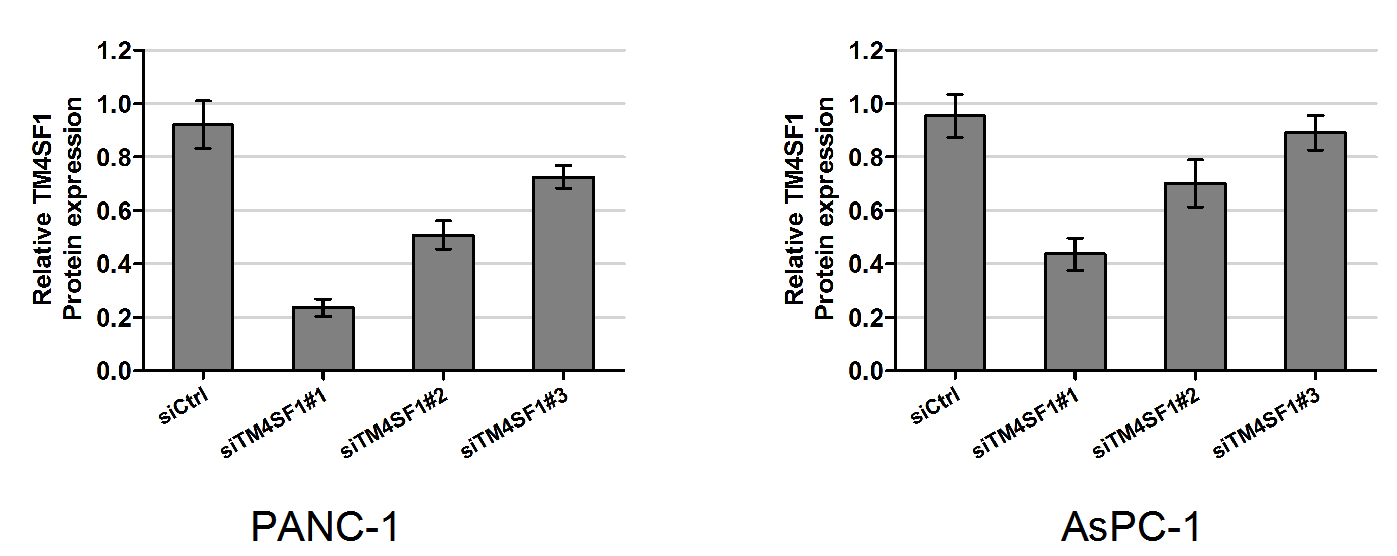


**Supplementary Figure S2.** **DDR1 over-expression rescues the inhibitory effects by TM4SF1 silenced on invadopodia formation.** **(A,B)** PANC-1 cells in the different conditions were stained with Tks5, F-actin and DAPI. Knockdown of TM4SF1 reduced the ability to form invadopodia significantly in PANC-1. And after overexpressing of DDR1 in TM4SF1 silencing cells, the number of cells with invadopodia increased.


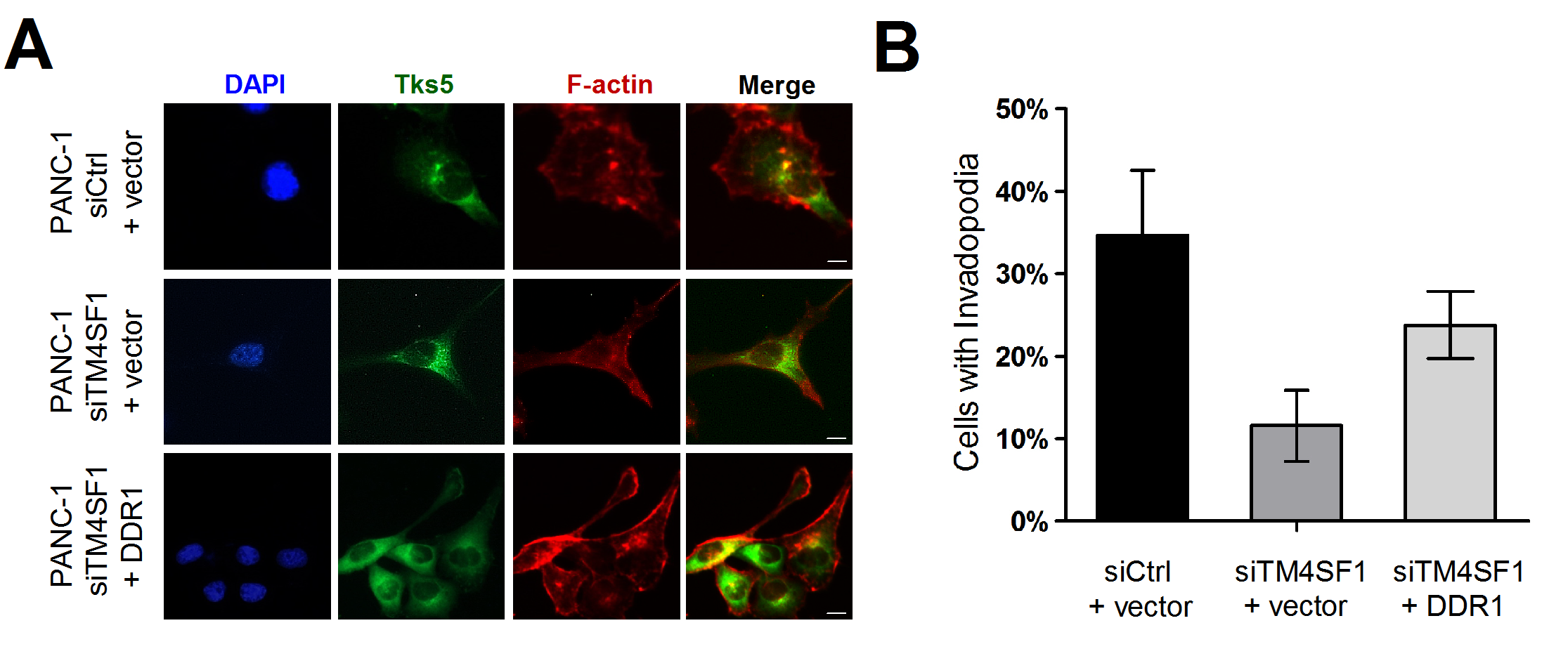

Supplement: Supplementary Figures [file srep45895-s1.doc]
